# Recovering the Original Intentions of Risk Assessment and Management of Genetically Modified Organisms in the European Union

**DOI:** 10.3389/fbioe.2018.00052

**Published:** 2018-05-04

**Authors:** Dennis Eriksson

**Affiliations:** Department of Plant Breeding, Swedish University of Agricultural Sciences, Alnarp, Sweden

**Keywords:** GMO, EU, risk assessment, risk management, regulation

## Early drafting of the GMO regulations

Progress in the field of recombinant nucleic acid techniques and cross-species gene delivery in the 1970s and 1980s prompted legislators in the European Union (EU) to develop biosafety regulations encompassing these techniques and their resulting products. The ensuing procedure for risk assessment and risk management of genetically modified organisms (GMOs), as these were denominated, in the EU has thus been established with the purpose of ensuring a high level of protection of human health and the environment. The early draft legislative texts on GMOs in the EU (Commission of the European Communities, 1988) resulted in the first Council Directive on the deliberate release into the environment of GMOs (Dir 90/220/EEC) in 1990 (Official Journal of the European Communities, 1990). From these early drafts, it is clear that the intentions were to have an evolving and increasingly trait-oriented regulatory framework taking into account technical developments, potential safe history of use as well as potential benefits resulting from the application of these techniques and their resulting products. However, despite nearly three decades of research, product development, demonstrated benefits and a lack of demonstrated risks associated with recombinant nucleic acids *per se*, the GMO regulatory framework in the EU has neither evolved nor been implemented as intended. I here list four details for which policy makers in the EU need to consider the original intentions of the GMO regulatory framework in order to correctly interpret the current legislative texts as well as allow for necessary updates following technical progress.

### Shifting focus to organisms and their traits

There is much unnecessary confusion nowadays on whether the EU is regulating GMOs on basis of the techniques that were applied or on the nature of the resulting organisms and their derived products. According to the EU Directive 2001/18/EC, a GMO is defined as “*an organism, with the exception of human beings, in which the genetic material has been altered in a way that does not occur naturally by mating and/or natural recombination”* (Official Journal of the European Communities, 2001). Custers (2017) points out that this definition is somewhat ambiguous regarding the interpretation of “altered in a way,” and shows that Annex 1A, part 1, of the same Directive gives further indications by stating that “*Techniques of genetic modification referred to in Article 2(2)(a) are inter alia: (1) recombinant nucleic acid techniques involving the formation of new combinations of genetic material […] and their incorporation into a host organism in which they do not naturally occur.”* This means that within the EU regulatory framework a GMO is achieved only when the application of a particular technique leads to a particular result, i.e., an organism carrying artificially recombined nucleic acids in novel formation. This view is also shared by other authors (Sprink et al., 2016a,b; Kahrmann et al., 2017) as well as by the European Commission (European Parliament, 2014).

If we go back to the early legislative drafts, it is clear that the intention has always been to regulate the resulting organisms and their derived products as much as, or perhaps to an even higher degree than, the underlying techniques. In the 1988 proposal for a Council Directive on the deliberate release to the environment of genetically modified organisms (GMOs), it was suggested that “*The present approach, which focusses on the new techniques of genetic engineering, is the first and most urgent step in the regulatory process; however, this will not impede evolution towards a more organism-related approach*”. Along with this aspiration, it was also noted that “*different categories of organisms and/or techniques may be established, allowing different requirements for organisms of different levels of risks*” (Commission of the European Communities, 1988). Models for setting up different risk-based categories of organisms and/or techniques have already been developed (Barton et al., 1997; Miller, 2010; Beker et al., 2016; Conko et al., 2016; Ricroch et al., 2016), whereas the EU is currently in practice arguably very far away from living up to these original intentions. Zetterberg and Edvardsson Björnberg (2017) have also recently suggested that a new protocol for risk assessment incorporating selected aspects of traits and gene functions, rather than the mere presence of recombinant nucleic acids in the product, may contribute to making the EU GMO legislation more consistent regarding criteria such as non-discrimination of techniques and scientific adaptability taking the latest scientific findings into account. In this context, it is also worth noting that the Norwegian Biotechnology Advisory Board has initiated a public discussion on the future regulation of gene technologies, asking if there is a need for new dividing lines (Norwegian Biotechnology Advisory Board, 2018). Norway is not an EU member state but, being part of the European Economic Area (EEA), has implemented the EU Directive 2001/18/EC in its Gene Technology Act (GTA). A level-based approval system is now being suggested for the discussion, based on the type and extent of genetic change. Other criteria are also relevant, including altered traits, the intended use of the organism, the risk to health or the environment, sustainability, societal benefits and ethics.

### Periodical updating of annexes

On 1st September 2017, the Netherlands published a proposal to improve the exemption mechanism for GM plants under Directive 2001/18/EC.^1^ This proposal was put forward to resolve the decade-long issue of the regulatory status of new plant breeding techniques (NPBTs), and suggests to amend Annex 1B of Directive 2001/18/EC which lists GM techniques yielding organisms that are excluded from the Directive. The amendment would add a list of criteria, exempting from regulation plants that (1) do not contain other genetic material than from the same, or a crossable, species and (2) do not contain recombinant nucleic acids. Not in any way redefining the logic of Directive 2001/18/EC, the proposal from the Netherlands is a congruent way to clarify the GMO definition, including what is regulated and what is not, beyond any reasonable doubt and provide a practical solution to handle certain NPBTs.

It is also perfectly aligned with the original intentions for the GMO regulatory framework in the EU. The 1988 proposal for a Council Directive advertises “*the commitment to update the Directive to technical progress as necessary, given the rapid scientific development of this field*” and declares further that “*the Commission shall adapt the annexes of this Directive to technical progress by amending new techniques to be covered or deleting as appropriate*” (Commission of the European Communities, 1988). However, the provision to amend the Annex listing techniques that yield or do not yield GMOs was not included in later Directives (Official Journal of the European Communities, 1990, 2001). However, nearly 30 years of technical progress is arguably a compelling reason to endow the current Directive with such a mechanism and the proposal from the Netherlands also suggests that a review process for periodical adaptations to technological progress should be designed.

The idea has been up for discussion before. In 2006, a research team from Wageningen University and Research Centre in the Netherlands proposed to add cisgenesis to Annex 1B of Directive 2001/18/EC on the grounds that the resulting plants are similar to traditionally bred plants (Schouten et al., 2006a,b). Though being criticized for not approaching the issue of whether or not the phylogenetic distance of donor and recipient organism is relevant to risk assessment (Giddings, 2006; Eriksson et al., 2014), it nevertheless provides important clues to the regulatory status of NPBTs given the current GMO regulatory framework in the EU and the definition contained therein.

### Acknowledging the history of safe use

Several GMOs and their derived products have been on the market in many countries and regions, including the EU, for more than two decades and these specific applications now arguably have a long safety record. Several reviews on GMO safety research demonstrates that no significant hazards directly associated with the use of recombinant nucleic acid techniques have been detected so far (Domingo and Giné Bordonaba, 2011; DeFrancesco, 2013; Nicolia et al., 2013). The International Centre for Genetic Engineering and Biotechnology (ICGEB) also provides a comprehensive collection of many thousands of scientific articles published since 1990 on biosafety and risk assessment in biotechnology.^2^

Directive 2001/18/EC states that its provisions should not apply to organisms which have conventionally been used and have a long safety record, however the same Directive lacks any indication of how to apply this very criterion of “long safety record” (Official Journal of the European Communities, 2001). The intention to take a long safety record into account was present already in the drafting of the first GMO legislation in the EU: “*The techniques not covered are those that have long been used with crop plants and livestock with an excellent safety record*”. That recombinant nucleic acid techniques were relatively new and untested in the 1980s was also emphasized several times: “*In a largely unexplored field like this, the exchange of information is likely to play an essential role in gaining experience*” (Commission of the European Communities, 1988).

Let us compare with conventional induced mutation breeding, which started to be applied large-scale in the 1950s (Oladosu et al., 2015). When the first GMO legislation in the EU was being developed in the late 1980s, induced mutations thus had a history of safe use stretching over more than 30 years and were therefore exempt from the regulatory provisions applied to GMOs. Today, GMOs have been used safely in commercial applications for more than 20 years and scrutinized in research and through market authorization requirements already significantly more than induced mutation breeding. One may therefore ponder the rhetorical question put by DeFrancesco (2013): “*How safe does transgenic food need to be ?*”. We now have plenty of evidence that recombinant nucleic acid techniques are not inherently unsafe and the responsible policy makers should therefore consider to modify the regulatory requirements accordingly, in part by shifting focus to organisms and their traits and also to initiate discussions on how to implement a model with risk categories based on traits and/or techniques as mentioned above.

### Acknowledging potential benefits

Plenty of reviews and meta-analyses have demonstrated the environmental, agricultural and economic benefits of certain GMOs and their derived products (Qaim, 2009; Fagerström and Wibe, 2012; Green, 2012; Mannion and Morse, 2012; Klümper and Qaim, 2014; Brookes and Barfoot, 2017). The 1988 proposal for a Council Directive on GMOs predicted the potential for these benefits and added that “*It must also be acknowledged that the use of GMOs could lead to improvements in health and the environment by permitting the development of more precise agricultural inputs for protection and nutrition*” (Commission of the European Communities, 1988). However, this acknowledgement is absent from the later Directives (Official Journal of the European Communities, 1990; 2001). Part of the general provisions of the current Directive 2001/18/EC is though that “*In accordance with the precautionary principle, the objective of this Directive is to approximate the laws, regulations and administrative provisions of the Member States and to protect human health and the environment*” (Official Journal of the European Communities, 2001). In light of the overwhelming evidence of benefits demonstrated by the references listed above as well as the absence of associated risks, it can be argued that certain applications of GMOs and/or their derived products are compatible with an interpretation of the precautionary principle that would promote, rather than prohibit, these applications. Returning to the analysis by Zetterberg and Edvardsson Björnberg (2017), they also suggest an alternative regulatory model based on sustainability criteria that apply to all varieties regardless of the applied breeding methods. This model would certainly be compatible with the precautionary principle as the primary goal would not be to merely avoid risk by refraining from the use of certain techniques but instead to achieve a broader set of sustainability goals.

## Conclusions

I have here argued that the regulatory framework for GMOs in the EU, and its implementation, has deviated considerably from the original intentions three decades ago when the fields of recombinant nucleic acid techniques and cross-species gene transfer were still relatively new in research and commercial applications. Given the experienced benefits of GMO applications, the safe history of use and the technical progress in the field of gene technologies, it is imperative to bring the GMO regulatory framework back in line with the original intentions and provide for a more trait- and benefit-oriented interpretation. The four above listed details provide starting points for discussions among policy makers in the EU.

## Author contributions

The author confirms being the sole contributor of this work and approved it for publication.

### Conflict of interest statement

The author declares that the research was conducted in the absence of any commercial or financial relationships that could be construed as a potential conflict of interest.

## Footnotes

^1^https://www.rijksoverheid.nl/binaries/rijksoverheid/documenten/kamerstukken/2017/09/13/proposal-for-discussion/proposal-for-discussion.pdf

^2^http://bibliosafety.icgeb.org/
